# BMP4 Increases the Expression of TRPC and Basal [Ca^2+^]_i_ via the p38MAPK and ERK1/2 Pathways Independent of BMPRII in PASMCs

**DOI:** 10.1371/journal.pone.0112695

**Published:** 2014-12-02

**Authors:** Yi Zhang, Yingfeng Wang, Kai Yang, Lichun Tian, Xin Fu, Yan Wang, Yueqian Sun, Qian Jiang, Wenju Lu, Jian Wang

**Affiliations:** 1 State Key Laboratory of Respiratory Diseases, Guangzhou Institute of Respiratory Disease, Guangzhou Medical University, Guangzhou, Guangdong, China; 2 Department of Laboratory Medicine, The 1st Affiliated Hospital of Guangzhou Medical University, Guangzhou, Guangdong, China; 3 The 2nd Affiliated Hospital of Guangzhou Medical University, Guangzhou, Guangdong, China; 4 Division of Pulmonary & Critical Care Medicine, Johns Hopkins University School of Medicine, Baltimore, Maryland, United States of America; 5 Department of Arts and Science, University of Toronto, Toronto, Ontario, Canada; 6 Department of Pulmonary, Inner Mongolia People's Hospital, Hohhot, Inner Mongolia, China; Vanderbilt University Medical Center, United States of America

## Abstract

Multiple abnormalities of bone morphogenetic protein (BMPs) signaling are implicated in the process of pulmonary arterial hypertension (PAH). BMP4 plays an important role during the process of pulmonary arterial remodeling and mutant of the principle BMP4 receptor, BMP receptors II (BMPRII), is found to associate with the development of PAH. However, the likely mechanism defining the contribution of BMPRII to BMP4 mediated signaling in pulmonary arterial smooth muscle cells (PASMCs) remains comprehensively unclear. We previously found that enhanced store operated calcium entry (SOCE) and basal intracellular calcium concentration [Ca^2+^]_i_ were induced by BMP4 via upregulation of TRPC1, 4 and 6 expression in PASMCs, and that BMP4 modulated TRPC channel expression through activating p38MAPK and ERK1/2 signaling pathways. In this study, BMPRII siRNA was used to knockdown BMPRII expression to investigate whether BMP4 upregulates the expression of TRPC and activating Smad1/5/8, ERK1/2 and p38MAPK pathway via BMPRII in distal PASMCs. Our results showed that knockdown of BMPRII: 1) attenuated BMP4 induced activation of P-Smad1/5/8, without altering BMP4 induced P-p38MAPK and P-ERK1/2 activation in PASMCs; 2) did not attenuate the BMP4-induced TRPC1, 4 and 6 expression; 3) did not affect BMP4-enhanced SOCE and basal [Ca^2+^]_i_. Thus, we concluded that BMP4 activated Smad1/5/8 pathway is BMPRII-dependent, while the BMP4 – ERK/p-P38 – TRPC – SOCE signaling axis are likely mediated through other receptor rather than BMPRII.

## Introduction

Pulmonary arterial hypertension (PAH) is a severe and progressive disease, with sustained elevation of pulmonary arterial pressure and a poor prognosis. Considerable studies have been focused on understanding the mechanisms of PAH, yet the underlying mechanisms have not been comprehensively understood. Recent advance found that abnormal bone morphogenetic proteins (BMPs) signaling deregulated the cell growth and differentiation, and contributed to pulmonary artery remodeling in the process of PAH [1], [2], [3]. Furthermore, substantial evidence indicates that BMP signals are important mediators in calcification of the intima and tunica media [4], which may lead to severe PAH [5].

BMPs belong to the TGF-β superfamily, which bind and activate heteromeric complexes of type I and type II receptors, while BMP signals regulate through binding the complex of type receptor I (ALK2, BMPRIa and BMPRIb) and type receptor II (BMPRII, ActRIIa and ActRIIb). Among these receptors, BMPRII is the most common single culprit gene. The expression of BMPRII significantly decreased in pulmonary artery isolated from patients with primary PAH and in some animal models of PAH induced by monocrotaline, chronic hypoxia or transgenic mice (SM22-rtTA X TetO7-MPR2^delx4+^) [6], [7], [8], [9], [10]. Moreover, it is generally accepted that mutations in the gene encoding BMPRII are responsible for patients with PAH and BMPRII expression is also decreased in PAH patients without BMPRII mutations [11], indicating that BMPRII signaling plays a crucial role in the development of PAH. In contrast, BMPRII-targeted therapy significantly reduced pulmonary arterial pressure, right ventricular hypertrophy and muscularization of distal pulmonary arteriolesin by upregulation of Smad signaling and reduction cell proliferation under chronic hypoxia [12]. More and more efforts have been devoted to understand how loss of BMPRII in the pulmonary vasculature contributes to enhanced vasoconstriction and vessel remodeling [13].

BMP4, a member of the BMPs, was recently considered as an important factor mediating the process of pulmonary arterial remodeling, got involved in the regulation of cell proliferation, migration, intracellular calcium homeostasis [1], [8], [14]. In common with other BMPs, BMP4 transduces its signal through binding to type I and type II receptors, which activates serine/threonine kinases, leading to transcriptional regulation by phosphorylating Smads (Smad1/5/8) [15], [16]. In addition to Smads pathway, mitogen-activated protein kinases (MAPKs) such as p38 or ERK1/2 can also be activated by BMP4 via Smad-independent mechanisms [17], [18], [19]. BMPRII is usually identified as the principle type II receptor that responds to BMP4. Although it has been suggested that dysfunctional BMP4 signal regulation on cell differentiation contribute to abnormal vascular remodeling, the critical importance of both BMP4 and BMPRII to these functions is poorly understood.

Our previous data showed that BMP4 treatment could enhance SOCE and basal [Ca^2+^]_i_ via upregulation of TRPC1, 4 and 6 expression in rat distal pulmonary artery smooth muscle cells (PASMCs) [20]. We also found that BMP4 may participate in the regulation of Ca^2+^ signaling by modulating TRPC channel expression through activating p38MAPK and ERK1/2 pathways. In this study, we investigated the role of BMPRII in the upregulation of TRPC expression, enhancement of calcium signing and activation of Smad1/5/8, ERK1/2 and p38MAPK pathway induced by BMP4 in distal PASMCs.

## Materials and Methods

### Animal protocol and Cell culture

In this study, the animal protocols were approved by the Animal Care and Use Committee of The Johns Hopkins University School of Medicine. Primary PASMCs were isolated and cultured from rat intrapulmonary arteries according to previous methods [21]. Briefly, adult male Wistar rats (200 to 350 g) were selected and anesthetized with pentobarbital sodium (65 mg/kg intraperitoneal injection). Distal intrapulmonary arteries (PA, >4^th^ generation) were dissected from lungs of rats. Adventitia and endothelium were removed from isolated PA. PASMCs were harvested from these vessels enzymatically and cultured for 3–4 days in smooth muscle growth media (SMGM-2, Clonetics), supplemented with 5% fetal bovine serum (FBS), 1% streptomycin, and 1% penicillin. Cellular purity was >90%, as evaluated by morphological appearance under phase-contrast microscopy and immunofluorescence staining for α-actin under confocal microscopy.

### Small interference RNA transfection

BMPRII small interference RNA (siRNA accession no. XM_217409) were designed and synthesized by Dharmacon (Lafayette, CO). Nontargeting siRNA served as a control. Transfection reagent obtained from GeneSilencer (Genlantis) was used according to the manufacturer's instructions. PASMCs at 50–60% confluence were transfected with 50 nM siRNA for 6 h in serum and antibiotic-free SMBM, then cultured for 42 h in SMBM with 0.3% FBS, followed by treatment with BMP4 at different time point.

### BMP4 treatment

PASMCs were transfected with BMPRII siRNA for 48 h, followed by treatment with 50 ng/ml recombinant human BMP4 (rhBMP4, R&D systems) for 10 min or 60 h respectively, before calcium image analysis and gene expression measurements.

### RNA extraction and real-time quantitative PCR

Total RNA was extracted using RNeasy kit (Qiagen, Valencia, CA) and reverse transcription was performed using iScript cDNA synthesis kit (Bio-Rad, Hercules, CA) according to the manufacturer's instructions. Primer sequences of rat TRPC1, 4, 6 are applied according to previous described [20] as TRPC1: sense 5′-AGCCTCTTGACAAACGAGGA-3′, anti-sense 5′-ACCTGACATCTGTCCGAACC-3′; TRPC4: sense 5′-GACACGGAGTTCCAGAGAGC-3′, anti-sense 5′-GTTGGGCTGAGCAACAAACT-3′; TRPC6: sense 5′-TACTGGTGTGCTCCTTGCAG-3′, anti-sense 5′-GAGCTTGGTGCCTTCAAATC-3′; Cyclophilin B: sense 5′-CAAGACCTCCTGGCTAGACG-3', anti-sense 5′-TTCTCCACCTTCCGTACCAC-3'; 18s (Rn_Rnr1_1_SG QuantiTect Primer Assay) were from Qiagen. SYBR green real-time PCR amplification was implemented using the iCycler IQ (Bio-Rad) system.

### Western blot analysis

PASMCs were lysed with ice-cold T-PER buffer (Pierce, Rockford IL) supplemented with protease inhibitors. The qualifications of cell lysate were analyzed using bicinchoninic acid protein assay (Pierce, Rockford, IL). Approximately 30 µg of protein were separated on 8% SDS-PAGE and then transferred to PVDF membranes (Bio-Rad). The membranes were blocked by 5% nonfat milk (Bio-Rad) with 0.2% Tween-20, then incubated with the following primary antibodies: anti-TRPC1, anti-TRPC4, anti-TRPC6, anti-phospho-Smad1/5/8, anti-phospho-ERK1/2, anti-phospho-p38MAPK, anti-ERK1/2, anti-Smad5, anti-p38MAPK, anti-BMPRIa, anti-BMPRIb, anti-ALK2, anti-BMPRII, anti-ActR2a, anti-ActR2b and anti-α-actin, respectively. Second antibodies were blotted with horseradish peroxidase-conjugated anti-rabbit, anti-mouse or anti-goat IgG (Kirkegaard and Perry Laboratories, Gaithersburg, MD). Band was detected by ECL (GE healthcare, Piscataway, NJ).

### Measurement of [Ca^2+^]_i_

The method of measuring [Ca^2+^]_i_ was present previously [22]. Briefly, [Ca^2+^]_i_ was determined with Fura-2 AM (Invitrogen) fluorescence emitted at 510 nm after excitation at 340 and 380 nm every 12 sec intervals. PASMCs loaded with 7.5 µM Fura-2 AM were incubated for 60 min at 37°C, then mounted in a closed polycarbonate chamber and perfused with Krebs-Ringer bicarbonate solution for 10 min (KRBS, 1 ml/min), which consisted of (in mM) 118 NaCl, 4.7 KCl, 2.5 CaCl_2_, 0.57 MgSO_2_, 1.18 KH_2_PO_4_, 25 NaHCO_3_, and 10 glucose. Changes in [Ca^2+^]_i_ were assessed with a Nikon TSE 100 Ellipse inverted microscope (Nikon, Melville, NY). Chamber temperature was maintained at 37°C with an in-line heat exchanger and dual-channel heater controller (models SF-28 and TC-344B, Warner Instruments). Data on changes in F340/F380 (Fura-2) were analyzed with InCyte software (Intracellular Imaging, Cincinnati, OH). [Ca^2+^]_i_ is presented as an average from 20–30 cells.

### Measurement of SOCE

Mn^2+^ quenching of Fura-2 AM fluorescence was used to evaluated SOCE as described previously [22]. Briefly, PASMCs were perfused for 10 min with Ca^2+^-free KRBS containing 1 mM EGTA to chelate residual Ca^2+^, 5 µM nifedipine to block calcium entry through L-type VDCC and 10 µM cyclopiazonic acid (CPA) to deplete SR Ca^2+^ stores. Fura-2 AM fluorescence excited at 360 nm every 30 sec was recorded for 5 min with Ca^2+^-free KRBS containing 1 mM EGTA and 10 min Ca^2+^-free KRBS with 200 µM MnCl_2_. SOCE was evaluated as the rate of decrease in Fura-2 AM fluorescence measured at excitation wavelength of 360 nm.

### Drugs and materials

Unless otherwise noted, all reagents were obtained from Sigma-Aldrich. All antibodies were obtained as follow: TRPC1, 6 antibodies (abcam), TRPC4 antibody (Alomone Laboratories), phospho-Smad1/5/8, phospho-ERK1/2, phospho-p38MAPK, ERK1/2, Smad5 and p38MAPK antibodies (Cell Signaling Technology), BMPRIa antibody (Proteintech Group), BMPRIb antibody (Invitrogen), ALK2 antibody (Novus), BMPRII antibody (BD), ActR2a and ActR2b antibodies (Abbiotec), α-actin antibody (Sigma-Aldrich).

Stock solutions of rhBMP4 at 50 µg/ml were made in 4 mM HCl containing 0.1% BSA. Stock solutions 30 mM of CPA and 5 mM nifedipine were made in dimethyl sulfoxide (DMSO) and 200 mM of MnCl_2_ was dissolvd in deionized water. Fura-2 AM was prepared as a 2.5 mM stock solution in DMSO containing 20% pluronic F-127 (Invitrogen).

### Statistical analyses

All data are reported as means±SEM. Statistical analyses were performed using Student's t-test or one-way analysis of variance (ANOVA). *P*<0.05 was considered as significant different.

## Results

### Knockdown of BMPRII specifically reduced BMPRII expression, without interacting similar structure BMP receptor BMPRIa in PASMCs

**Figure 1:**
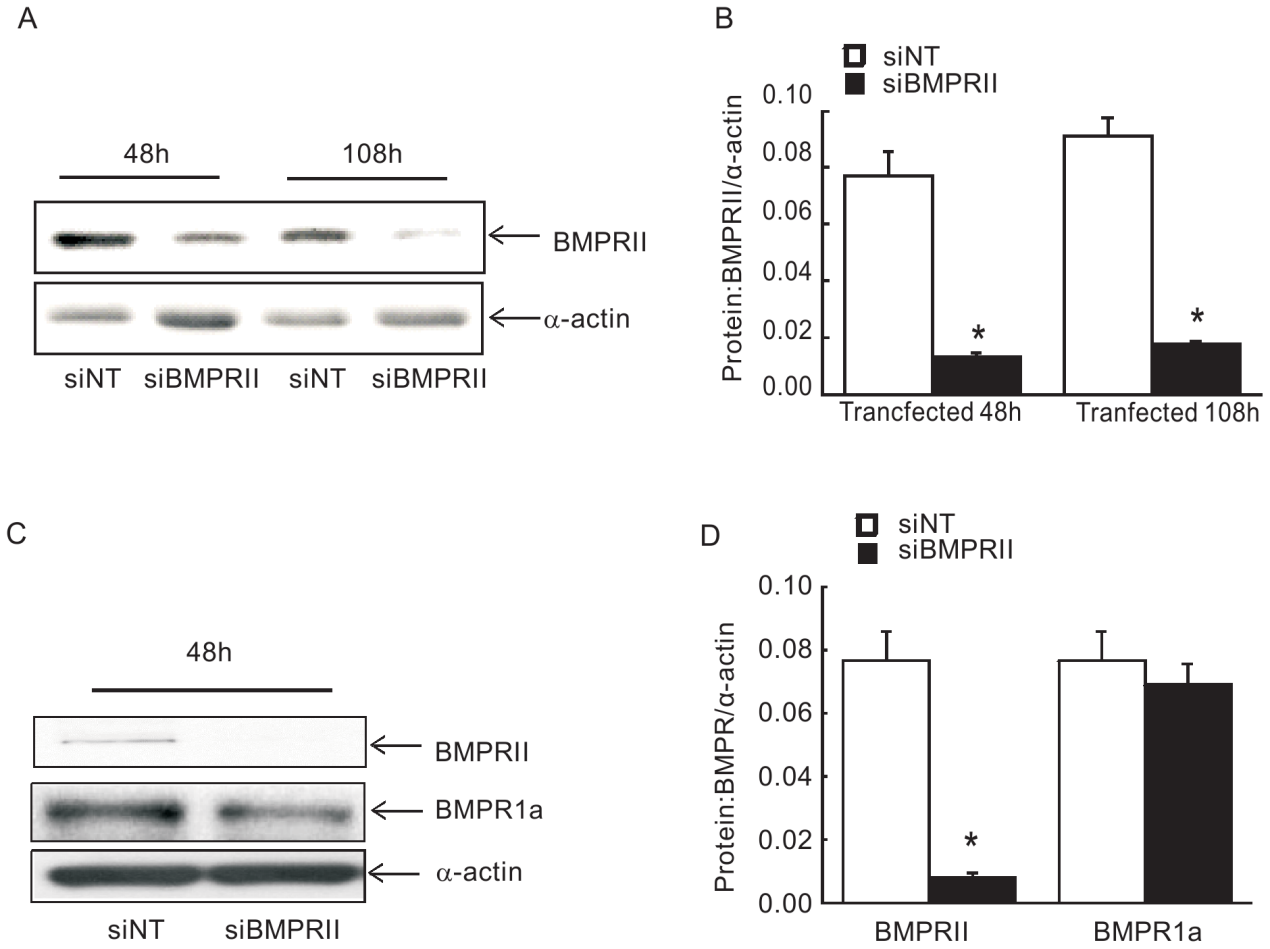
Specific siRNA effectively knockdown BMPRII protein without affecting BMPRIa protein in PASMCs. Images showing the blots of BMPRII protein for 48 h or 108 h (A) and BMPRIa protein relative to α-actin as detected by Western blotting in PASMCs treated with BMPRII small interference siRNA or non-targeting (NT) control siRNA for 48 h (C). Bar values are means ± SEM (n  =  4 for each group). *P <0.01 vs. respective siNT control (B and D).

To determine the role of BMPRII in PASMCs, we used siRNA targeted to BMPRII (BMPRII siRNA) to ablate BMPRII expression in PASMCs. The efficiency of knockdown of BMPRII was evaluated by Western-blot. The specificity of BMPRII siRNA was verified by expression levels of BMPRIa, which shares similar amino acid structure. As seen in Fig. 1A and B, BMPRII protein expression in BMPRII siRNA transfected cells was significantly decreased over 85% at 48 h or 108 h compared with non-targeted siRNA control, without altering expression of BMPRIa at 48 h (Fig. 1C and D), indicating the efficiency and specificity of endogenous BMPRII gene interference using siRNA.

### Ablation of BMPRII expression reduced P-Smad1/5/8, did not alter P-ERK1/2 and P-p38MAPK pathway expression induced by BMP4 in PASMCs

**Figure 2:**
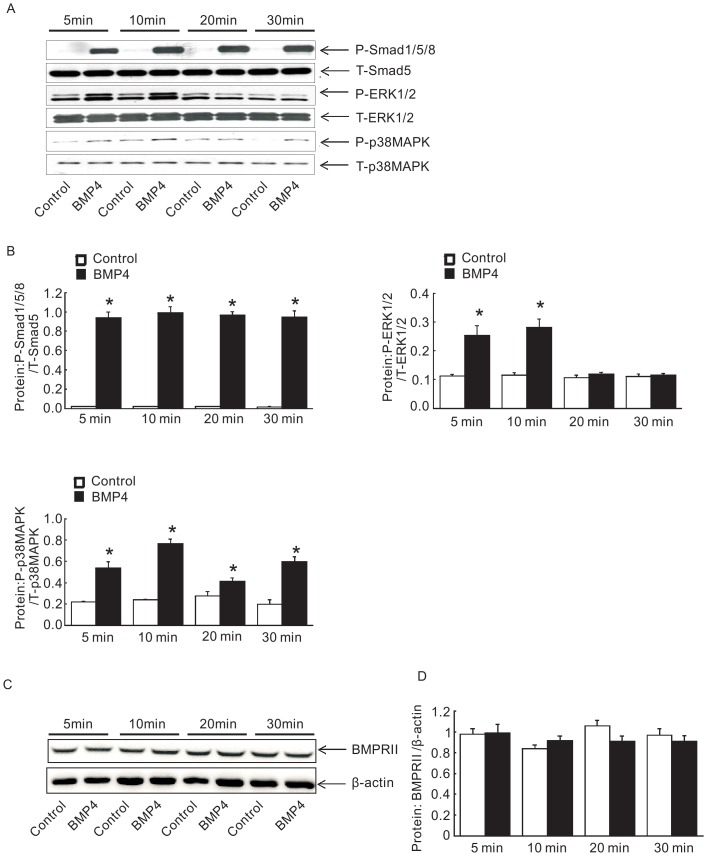
BMP4 induced activation of P-Smad1/5/8, P-p38MAPK and P-ERK1/2 in rat distal PASMC. (A) represents the blots of P-Smad1/5/8, P-p38MAPK, P-ERK1/2, t-Smad1/5/8, t-p38MAPK and t-ERK1/2 upon BMP4 treatment (50 ng/ml) in dose and time dependent manner. (B) Bar values are means ± SEM (n  =  3 for each group). (C) represents the blots of BMPRII upon BMP4 treatment for 5–30 min. (D) Bar values are means ± SEM (n  =  3 for each group).*P <0.01 vs. respective vehicle control.

**Figure 3:**
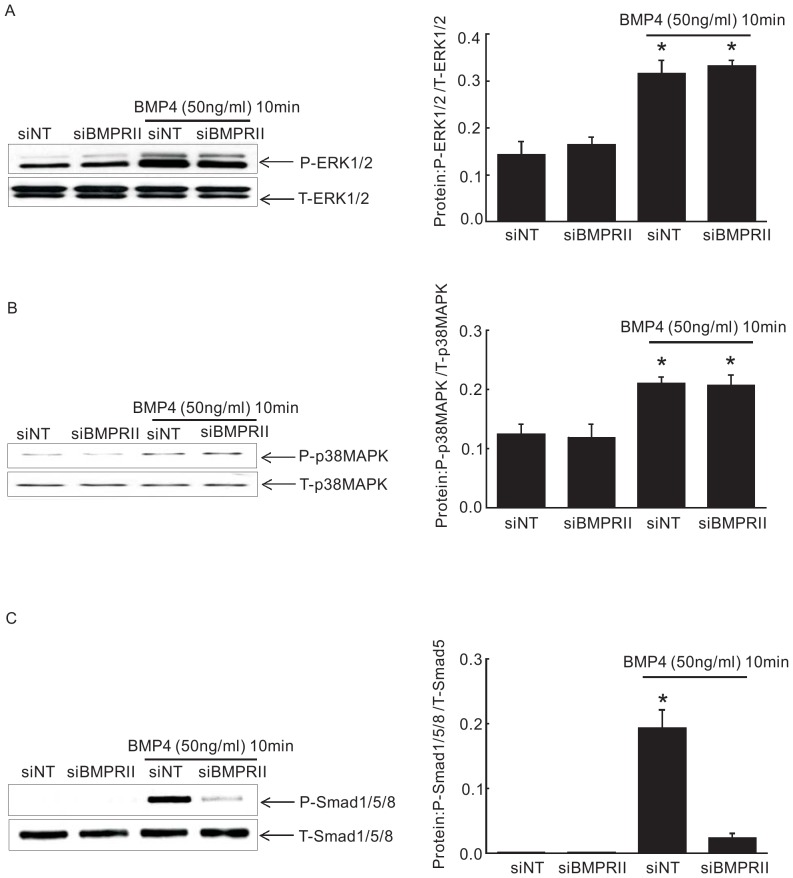
Knockdown of BMPRII attenuated BMP4 induced phosphorylation of P-Smad1/5/8, but not P-p38MAPK and P-ERK1/2 in PASMC. (A), (B) and (C) show blots (left) and normalized bar graphs (right) of P-Smad1/5/8, P-p38MAPK, P-ERK1/2, t-Smad1/5/8, t- p38MAPK and t-ERK1/2, respectively. Bar values were means ± SEM (n  =  4 for each group). *P <0.01 vs. respective siNT control.

As previously described in our early study, 50 ng/ml of BMP4 treatment has been determined to be the optimal dosage to regulate downstream gene expression [20]. Firstly, we evaluated the phosphorylation of Smad1/5/8, ERK1/2 and p38MAPK pathways in PASMCs at various time points from 5 min to 30 min following BMP4 (50 ng/ml) or vehicle treatment, as confirmed by Western blotting. Meantime, to determine whether short period of BMP4 treatment alone could induce BMPRII protein expression changes, we also detect the protein expression of BMPRII after BMP4 exposure at various time points from 5 min to 30 min. Our results showed that the phosphorylation of Smad1/5/8, p38MAPK and ERK1/2 was most obvious at 10 min of BMP4 treatment (Fig. 2A and B) but no change in the protein expression of BMPRII (Fig. 2C and D). Therefore, we focused on the 10-min time point. We next tested the effect of BMPRII loss on BMP4 induced Smad1/5/8, ERK1/2 and p38MAPK signaling activation using siBMPRII in PASMCs. Ablation of BMPRII significantly attenuated the BMP4 induced rapid phosphorylation of Smad1/5/8 without altering that of ERK1/2 and p38MAPK in PASMCs (Fig. 3A, B and C), indicating an indispensable role of BMPRII in BMP4 induced Smad 1/5/8 activation, but not ERK1/2 or p38MAPK.

### BMP4 induced TRPC1, TRPC4 and TRPC6 expression increase is not BMPRII dependent

**Figure 4:**
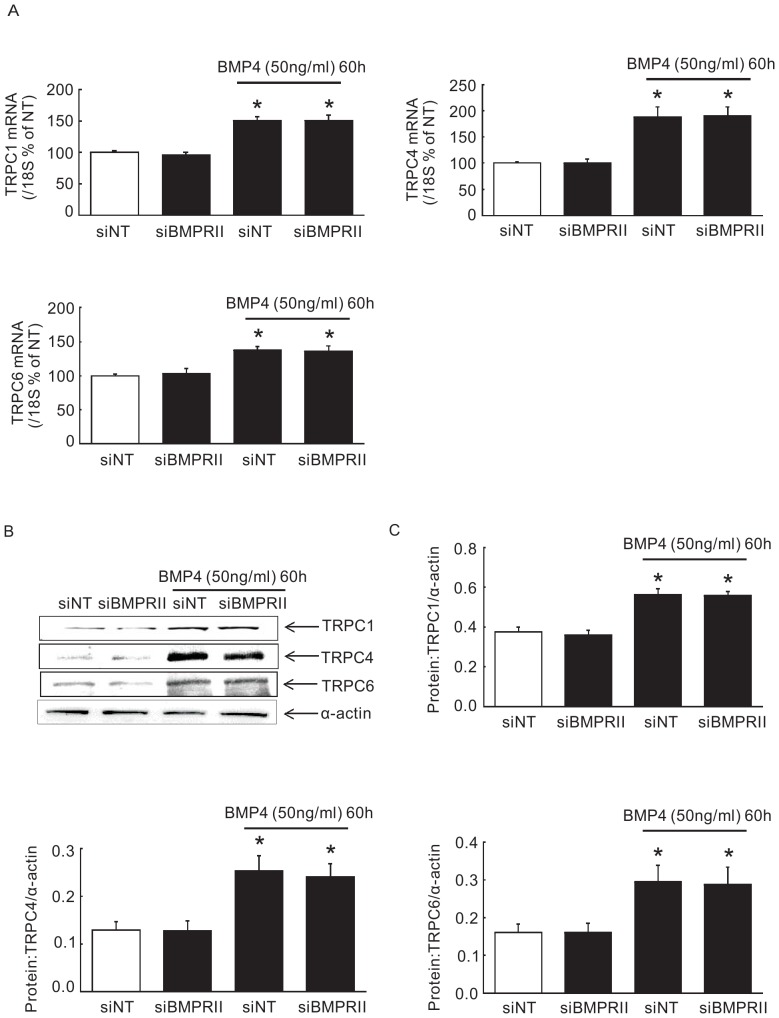
Knockdown of BMPRII did not affect BMP4 induced TRPC expression in PASMCs. (A) bar graphs showing the real-time PCR results indicating the mRNA expression of TRPC1, TRPC4 and TRPC6. *P <0.05 vs. respective vehicle control (A). (B) blots represent the protein expression of TRPC1, 4 and 6 after treatments of BMPRII and BMP4 and (C) represent the normalized bar graphs of TRPC1, TRPC4 and TRPC6 protein expression. Bar values are means ± SEM; *n*  =  3 in each group. §*P* <0.05 vs. respective siNT control.

We have previously found that 60 hours of BMP4 treatment enhanced mRNA and protein expression of TRPC1, TRPC4 and TRPC6 in PASMCs [20]. To assess whether BMP4 upregualtes the expression of TRPC1, TRPC4 and TRPC6 via BMPRII pathway in PASMCs, we interfered the endogenous BMPRII gene expression in PASMCs, followed by 60 hours of BMP4 treatment, and then detected the mRNA and protein expression of TRPC1, TRPC4, and TRPC6. We found that knocking down BMPRII expression did not alter the BMP4 induced expression of TRPC1, TRPC4 and TRPC6 mRNA and protein in PASMCs, indicating that TRPC1, TRPC4 and TRPC6 expression induced by BMP4 is not BMPRII dependent (Fig. 4).

### BMP4 induced SOCE and basal [Ca^2+^]_i_ enhancement is not BMPRII dependent

**Figure 5:**
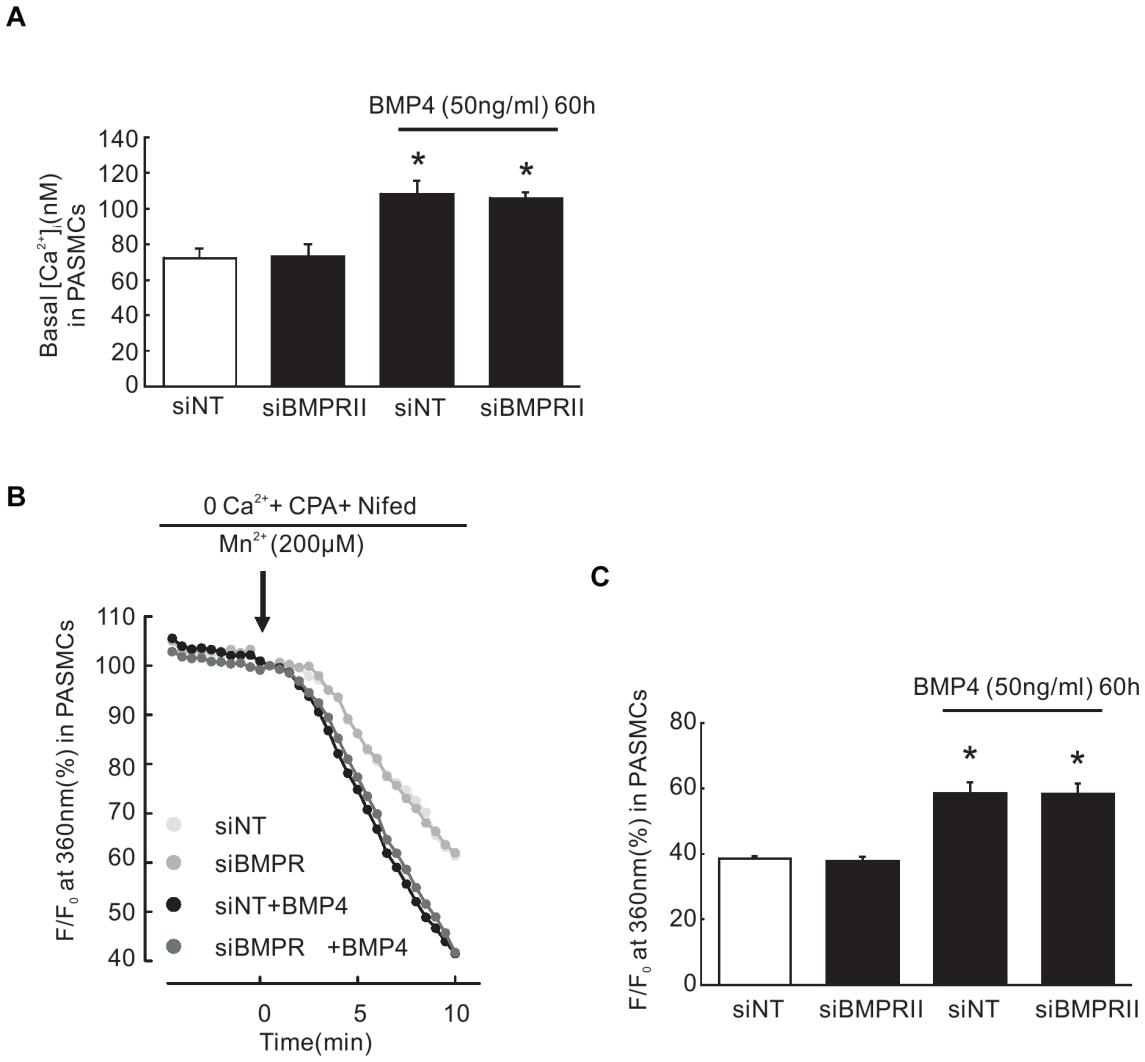
Knockdown of BMPRII did not affect BMP4 elevated basal [Ca^2+^]_i_ and SOCE in PASMCs. (A) bar graph showing the changes in basal [Ca^2+^]_i_ in distal PASMCs treated with 50 ng/ml BMP4 (n  =  4 from 120 cells) for 60 h. Bar values are means ± SEM. *P <0.05 vs. siNT control (n  =  4 from 120 cells). (B) represting time course of quenching of Fura-2 AM fluorescence at 360 nm by 200 µM Mn^2+^ after perfusion with Ca^2+^ -free KRBS (0 Ca^2+^) containing Nifedipine (5 µM) and CPA (10 µM) in distal PASMCs treated with 50 ng/ml BMP4 (n = 4 experiments in 110 cells) or vehicle for 60 h, normalized to fluorescence at time 0 (F/F_0_). (C) Bar graph shows that Mn^2+^ quenching, expressed as percent decrease in fluorescence at time 10 min from time 0, was greater in BMP4-treated distal PASMCs compared with that in control cells (*P<0.05). Bar values are means ± SEM.

Our group have already demonstrated that BMP4 could increase SOCE and basal [Ca^2+^]_i_ in PASMCs [20]. To testify whether BMP4 enhanced SOCE and basal [Ca^2+^]_i_ via BMPRII pathway in PASMCs, we interfered the endogenous BMPRII gene expression using siRNA, followed by 60 hours of BMP4 treatment, and measured the SOCE and basal [Ca^2+^]_i_ in PASMCs. Our results showed that knockdown of BMPRII expression did not affect BMP4 induced SOCE and basal [Ca^2+^]_i_ increases, indicated that BMP4 inducing SOCE and basal [Ca^2+^]_i_ increases is not mediated via BMPRII (Fig. 5).

### BMP4 treatment decreased BMPRII expression, enhanced ActR2a and ActR2b expression, without altering BMPRIa, BMPRIb, ALK2 in PASMCs

**Figure 6:**
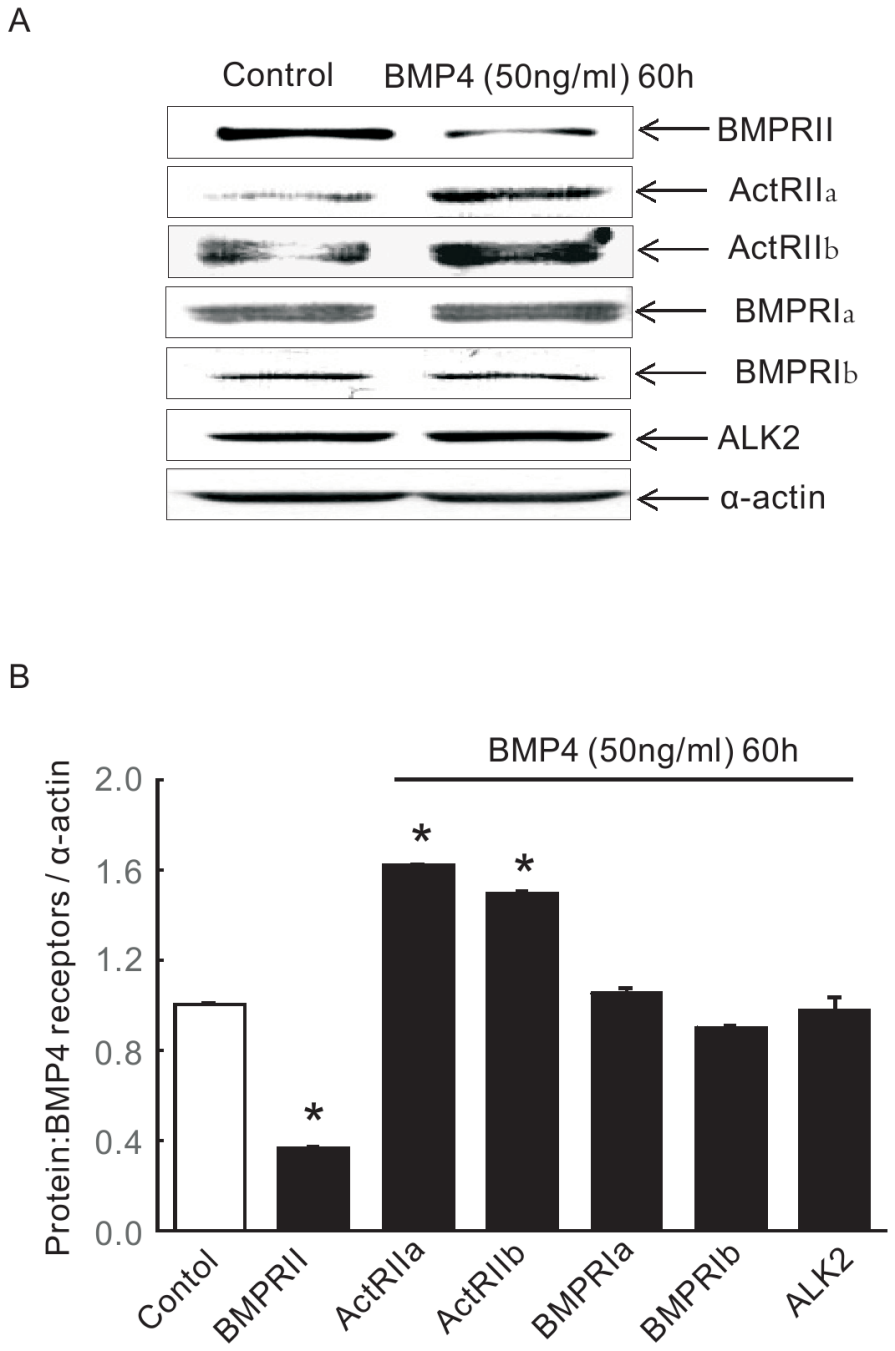
Expression profile of BMP receptors after BMP4 treatment in PASMCs. Representative blots (A) and normalized bar graphs (B) of BMPRII, ActRIIa, ActRIIb, ALK2, BMPRIa and BMPRIb protein as detected by Western blotting in PASMCs treated with 50 ng/ml BMP4 for 60 h. Bar graph shows mean protein expression for BMPRII, ActRIIa, ActRIIb, ALK2, BMPRIa and BMPRIb protein relative to α-actin (n  =  4 in each group; *P <0.05 vs. vehicle control). Bar values are as means ± SEM.

To explore the receptors involved in BMP4 induced SOCE and basal [Ca^2+^]_i_ enhancement, and TRPC1, −4, −6 expression increases, we tested the protein expression of type I (ALK2, BMPR1a and BMPR1b) and type II (BMPRII, ActRIIa and ActRIIb) receptors in BMP4 treated PASMCs. Compared with vehicle (control), BMP4 treatment for 60 h decreased the protein expression of BMPRII by 157.5%, enhanced the protein expression of ActR2a and ActR2b by 161.4% and 150% respectively, and did not lead to visible change in the expression of BMPRIa, BMPRIb, Alk2 in distal PASMCs (Fig. 6).

## Discussion

We have previously demonstrated that BMP4 increased TRPCs expression, SOCE and basal [Ca^2+^]_i_ in rat distal PASMCs (20). In the present study, BMPRII was found involved in BMP4 induced activation of Smad1/5/8, but not p38MAPK or ERK1/2. BMP4 increased TRPCs expression, SOCE and basal [Ca^2+^]_i_ was not BMPRII dependent. In addition, BMP4 treatment significantly decreased the expression of BMPRII, enhanced the expression of ActR2a and ActR2b, without visible change in the expression of BMPRIa, BMPRIb, ALK2 in distal PASMCs, suggesting the up-regulated ActR2a and ActR2b could be the potential candidates to mediate the elevated BMP4 signaling transduction involving the activation of p-ERK1/2 and p-P38MAPK.

Previous publication demonstrated that BMP4 signaling is mainly mediated by BMPRII, which is considered and recognized as the principal type II receptor in most cell lines [23], [24], [25], [26], [27]. BMP ligands lead to phosphorylation and activation of type I receptor kinases by constitutively activating type II receptor kinases. Activation of type I receptors caused phosphorylation and nuclear translocation of receptor-mediated Smads (R-Smad) (Smad 1/5/8). Intranuclear R-Smads then formed complexes with the common partner Smad (Co-Smad) Smad4 to bind and regulate the transcription of target genes [28], [29]. Missense mutations or haploinsufficiency of BMPRII can lead to deficiency of ability to transduce signals via Smads pathway [14], [23]. It has been reported at least two alternate pathways in response to BMP4, demonstrated as Smad dependent (Smad1/5/8) and Smad independent (ERK1/2 and p38MAPK) [17], [18], [19]. There is mounting evidence that MAPK signaling participates in Smads pathway regulation [30].

In this study, in order to figure out whether the BMP4 induced activation of all the three known downstream signaling transduction (p-Smad1/5/8, p-ERK1/2 and p-P38MAPK) are mediated by BMPRII, we firstly used BMPRII siRNA to specifically inhibit its endogenous gene expression. Both the efficiency and specificity of knockdown of BMPRII are qualified. Next, by using effective knockdown of BMPRII, we tested whether knockdown of BMPRII influenced activation of Smad1/5/8, ERK1/2 and p38MAPK downstream pathway induced by BMP4 in cultured rat distal PASMCs. We found that BMP4 induced rapid phosphorylation of Smad 1/5/8, but not ERK1/2 or p38MAPK, was attenuated by BMPRII ablation in PASMCs. These results suggest that BMP4 induced Smad1/5/8 pathway is activated via BMPRII. However, BMPRII was determined not involved in BMP4 induced ERK1/2 and p38MAPK pathway activation.

Intracellular Ca^2+^ is important for critical processes such as cell proliferation and apoptosis, and the changes in [Ca^2+^]_i_ are therefore considered to play a crucial role in the process of structure remodeling during the development of pulmonary hypertension [31], [32], [33], [34], [35]. We and others have previously found that SOCE was involved in the maintenance of [Ca^2+^]_i_ homeostasis and TRPCs were defined as the molecular components of SOCCs in PASMCs [21], [36], [37], [38]. In addition, our previous publication demonstrated that BMP4 enchances SOCE and basal [Ca^2+^]_i_ through upregulating TRPC expression [20]. We previously found that BMP4 up-regulated TRPC expression in distal PASMCs [20]. Hence, here we further determined whether TRPC1, TRPC4 and TRPC6 expression were up-regulated by BMP4 via BMPRII and the results showed that increased TRPC1, 4 and 6 expression induced by BMP4 is BMPRII-independent. It is consistent with our preliminary result which indicated that BMP4 modulate TRPC channel expression through p38MAPK and ERK1/2 pathways. Although Smad1/5/8 is generally considered as a key component of the canonical BMP signal transduction pathway, our data suggest that BMP4 induced TRPC1, 4 and 6 enhancement is dependent on p38MAPK and ERK1/2, but not Smad1/5/8. Recent studies demonstrated that abnormal expression of TRPC is an important factor in artery remodeling during PAH development [39], [40], [41], [42]. Proliferating PASMCs are found associated with increased TRPC1 expression [40]. In contrast, reduction of TRPC1 expression attenuates the proliferation of PASMCs [39]. Moreover, change in TRPC1 expression has been confirmed along with apoptosis [43].

Next, our results further showed that BMP4 could lead to enhancement of SOCE and basal [Ca^2+^]_i_ in the absence of BMPRII. The results are consistent with our above-mentioned findings, which indicate that BMPRII didn′t contribute to the regulation of Ca^2+^ signaling by modulating BMP4 induced TRPC channel expression.

Given the fact that BMPRII is unrelated to BMP4 induced elevated SOCE and basal [Ca^2+^]_i_, here came the hypothesis that other BMP receptors are potentially responsible for these subsequent outcomes of BMP4 treatment. Although people have obtained data suggesting that BMP4 mediate signaling through other candidates such as ALK2, BMPRIa, BMPRIb, ActRIIa and ActRIIb, the precise molecular mechanisms remain largely unknown. Many advances have confirmed that excision of BMPRII may drive BMP4 tend to combine with other type II receptors such as ActRII through utilizing coreceptor repulsive guidance molecule RGMa and result in the reduction of Smads pathway activation, without disrupting the activation of p38MAPK and ERK1/2 in PASMCs [17], [44], [45]. Based on these previous evidence, a further study was then undertaken to identify the expression profile of all the six known BMP receptors ActRII2a, ActRII2b, ALK2, BMPRIa and BMPRIb. We found that ActRIIa and ActRIIb were selectively up-regulated by BMP4 treatment. These results strongly suggest ActR2a and ActR2b mignt act as the potential candidates to charge for the excessive BMP4 induced downstream activation of p-ERK1/2 and p-P38MAPK signaling pathways, which then induced the elevated TRPC expression and enhanced SOCE and basal [Ca^2+^]_i_ in PASMCs. These works needs further evidence to elucidate in our future study.

In conclusion, this study confirmed that activation of Smad1/5/8 pathway by BMP4 is dependent on BMPRII, while BMP4 induced upregulation of TRPC expression, enhancement of calcium signaling and activation of ERK1/2 and p38MAPK pathway may depend on other receptors rather than BMPRII.
